# Quality of life impairment after a diagnosis of Cushing’s syndrome

**DOI:** 10.1007/s11102-022-01245-9

**Published:** 2022-06-29

**Authors:** Susan M Webb, Elena Valassi

**Affiliations:** 1 Endocrinology Dept, Hospital Sant Pau, IIB-Sant Pau, Research Center for Pituitary Diseases; Dept Medicine, Universitat Autònoma de Barcelona (UAB), CIBERER, Unit 747, ISCIII, 08041 Barcelona, Spain; 2 Endocrinology Dept, Germans Trias i Pujol Hospital & Research Institute, Universitat Internacional de Catalunya (UIC), CIBERER, Unit 747, ISCIII, 08916 Badalona, Spain

**Keywords:** Quality of life, Cushing syndrome, Memory impairment, Anxiety, Depression, Emotional distress

## Abstract

This brief review is devoted mainly to publications in the last 5 years dealing with health-related quality of life (QoL) after a diagnosis of endogenous hypercortisolism, due to pituitary-dependent Cushing’s disease (CD) or any other cause of Cushing syndrome (CS). Despite improvement after treatment, persistent physical morbidity, neurocognitive problems like worse executive capacity and memory as well as stress intolerance, depressive symptoms and more anxiety, lead to long-term impairment of QoL.

## Introduction

Ninety years ago, psychiatric and neurocognitive consequences of hypercortisolism were already described by Cushing in his original description of this syndrome [cited by 1]. In the last decades these most distressing neurocognitive complaints have been increasingly described, like loss of memory, difficulties in concentration, coping with emotions and with executive functions, intolerance to stress, emotional incontinence, irritability, anxiety and depression, that negatively impact on health-related quality of life (QoL), which despite improving after adequate therapy to control hypercortisolism, may not normalize [1–6]. Furthermore, other persisting problems like sleep disturbances, fatigue, muscle weakness and permanent weight gain contribute to explain why patients with “cured” Cushing disease (CD) or any kind of endogenous Cushing syndrome (CS) still do not feel well [2–7].

### Hypercortisolism impairs QoL

**Fig. 1:**
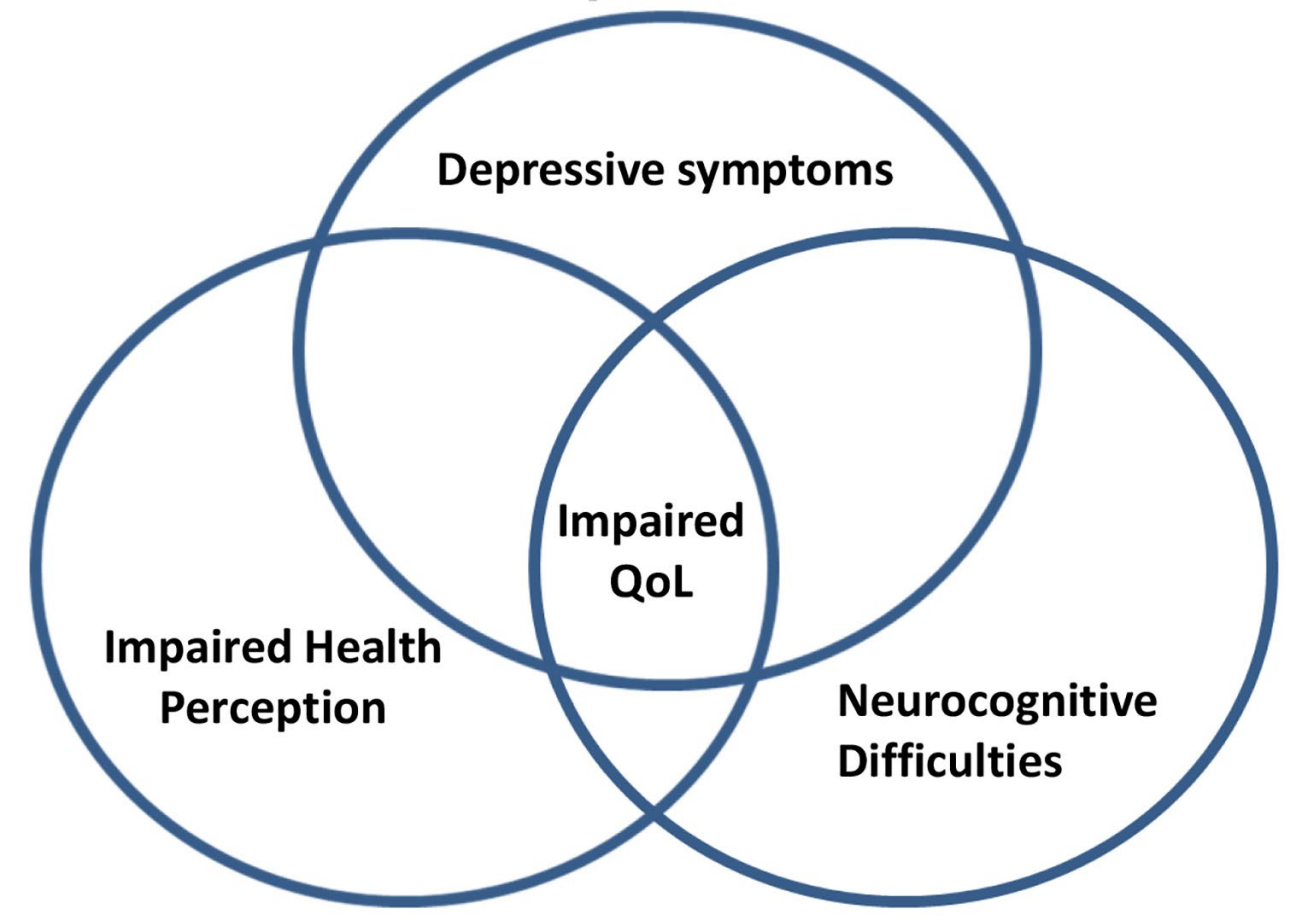
Impaired Quality of Life (QoL) in Cushing’s syndrome is determined by impaired health perception (due to persistent symptoms and morbidity), depressive symptoms and neurocognitive difficulties (including memory, executive functioning, intelligence and attention), leading to difficulties to cope with stress and emotions

Impaired QoL and cognitive functions (including attention and processing speed, visuospatial perception and construction, executive function, verbal learning and memory, visual learning and memory, verbal function and language skills, intelligence domain) are present at diagnosis in CS compared to healthy controls [1, 3]. QoL is equally affected by pituitary and adrenal causes of CS, on both the generic EuroQoL and specific CushingQoL questionnaire (more sensitive to Cushing-related dimensions). Postoperatively, cognitive function improves in most domains, as well as QoL, but patients often still complain of having less memory than before [8]. After treatment, CushingQoL scores are worse in pituitary than in adrenal patients, depending on the remission status, which more frequently occurs in the latter [8]. Additionally, older age and depression at diagnosis predict worse long-term outcome for QoL, regardless of aetiology. In fact, depressive symptoms, which negatively impact QoL, are present in most CS patients, either at baseline or during follow-up [3, 5]. The high prevalence of depression is also evidenced by the 2- to 4-fold higher use of antidepressants, anxiolytics and sleeping pills in CD than in controls, prior to and at diagnosis, and remains high for antidepressants and sleeping pills 5 years after diagnosis [9]. This is not surprising since after surgery patients expect hypercortisolism and all its symptoms and consequences to be resolved; they are told their hormones have normalized and yet they may feel worse than before, both physically and psychologically. Feeling helpless, bewildered, anxious and/or depressed is common, especially if in parallel they experience flare-ups of prior autoimmune conditions, carpal tunnel syndrome or generalized pain related to postoperative adrenal insufficiency and glucocorticoid deprivation [5] Fig. 1. Being unaware of what to expect by prior information and educational programs, may lead them to crash down and be unable to cope, especially in stressful situations [5]. Experiencing less memory and executive capacity compared to their prior condition, is also very distressing, as well as difficulty in coping with emotion; thus, being aware that this may occur and an empathetic attitude of their health providers is reassuring and helps to adapt to and cope with this new situation, benefiting health perception and QoL.

Five years after diagnosis and treatment for CS the use of anxiolytics decreases [9], probably reflecting improvement in perceived co-morbidities and/or an acceptance and adaptation to the “post-Cushing” situation. Nevertheless, CS patients still are prescribed three times more opioids as pain-killers compared to general population after 5 years of follow-up, but not anymore after 10 years. Interestingly, drug consumption 5 years after diagnosis did not differ among CD patients in remission or with persistent hypercortisolism, as far as antidepressants, anxiolytics, sleeping pills, opioids, renin angiotensin system blockers and antidiabetics, pointing out that morbidity is still increased despite endocrine cure. Thus, residual physical and neuropsychiatric morbidity in CS also entails a high socio-economic impact and leads to reduced employment status and increased requests for disability pensions [10].

Persistent muscle weakness, easy fatigability and tiredness are common complaints in treated CS, which greatly impact daily life and physical activity. Abnormal skeletal muscle structure and function persist after long-term remission of hypercortisolism, with more intramuscular fat mass, worse muscle performance and strength on physical testing and a greater prevalence of sarcopenia [4, 11]. Worse QoL is seen in “cured” CS patients compared to controls, especially if sarcopenia is present. They also experience more pain, easy bruising and worries on physical appearance in the CushingQoL questionnaire, and poorer physical functioning on the generic SF-36 questionnaire.

Neuroimaging in CS evidences persistent reduction of right hippocampal volume, grey matter volumes, cortical thickness, white matter integrity especially in structures important for cognitive function and emotional processing, and altered resting state functional responses during cognitive tasks [1]; a direct effect of chronic glucotoxicity on the central nervous system is thought to be involved in these structural and functional impairments, which often do not normalize despite remission of hypercortisolism.

### Impact of Cushing’s syndrome on QoL as evaluated by physicians

Perspectives on the recovery after treatment for CS are divergent between patients and physicians. While the desire of patients is to lead a normal life, without limitations or pain, for the physician a normalization of biochemical data is the main item to consider a “cure”. Physicians tend to underestimate the time to complete postoperative recovery, while patients with adrenal etiologies experience longer duration of adrenal insufficiency requiring cortisol replacement [12]. Physicians overestimate the benefits of work, exercise and activities, while patients find family/friends and rest to be helpful to cope with the complex postoperative period. Although when asked, both providers and patients consider that education on recovery is important, only one third considered to have received sufficient information from their physician for the postsurgical experience. Being empathetic and understanding the patients’ narrative of the experience of recovery is important, if QoL is to be improved. In this respect, nurses tend to be better than doctors, and are highly appreciated by the patients [13, 14].

### Patients’ perception on the impact on QoL after Cushing’s syndrome

It is highly frustrating for patients who may find no understanding in their managing health professionals, who consider them cured, and yet they certainly do not feel healthy. Awareness of these persistent physical and neuropsychiatric problems, as well as understanding, is essential, so that specific treatment can be offered; providing information so they are aware of these problems can be very therapeutic. Individualized educational programs for pituitary patients aimed at promoting autonomy and self-management improves QoL, satisfaction and health perception. New goals like reinforcing knowledge, sharing experiences, and improving self-confidence are observed [15]. Another self-management intervention program for pituitary patients and their partners approached psychosocial issues, like self-efficacy, bother and needs for support, illness perceptions, coping and QoL; results were positive when compared with a control group who did not follow the program. Improvements were seen in patient mood, self-efficacy, and vitality; partners reported less anxiety and depressive symptoms [16]. Patients should not blame themselves for not coping and be realistic for work expectancies, socially or at home [5].

Objective information from physicians on postsurgical recovery after CS, including duration, symptoms to expect, and helpful coping mechanisms are highly appreciated. In contrast, failing to understand a patient’s conception of recovery by health providers may lead the patient to feeling resentful. Fostering explicit education and discussions with the patients’ close environment, like partners and family, mentioning the need for increased social support, tends to lead to deeper discussion and understanding between physicians, patients, and their families, and ultimately improve QoL.

A further survey from 2 German neurosurgical referral centers and members of a US-based CS patient support group analyzed subjective illness distress and specific needs of supportive measures beyond medical interventions [13]. In both countries patients still suffered from Cushing-related symptoms, declared a reduced performance, and psychological problems. Good medical care and competent doctors helped most in coping with the illness. The majority, especially in the US, were interested in support groups and in courses on illness coping, or availability of information brochures. Most declared to be willing to attend internet-based programs. All needed skilled physicians and long-term medical care in dealing with the effects of CD/CS.

## Conclusions

Patient education and awareness of the physical and psychological aftermath in treated CS helps to overcome the postoperative period. Psychological interventions may be required to treat depression and to improve long-term prognosis, subjective wellbeing and QoL. Apart from benefiting the patients, their families, working and social capacity, it will reduce health costs.
